# Assessment of the association between increasing membrane pore size and endotoxin permeability using a novel experimental dialysis simulation set-up

**DOI:** 10.1186/s12882-017-0808-y

**Published:** 2018-01-05

**Authors:** Eva Schepers, Griet Glorieux, Sunny Eloot, Michael Hulko, Adriana Boschetti-de-Fierro, Werner Beck, Bernd Krause, Wim Van Biesen

**Affiliations:** 1 Department of Internal Medicine, Nephrology Division, Ghent University Hospital, Gent, Belgium; 2 Baxter International Inc., R&D, Hechingen, Germany

**Keywords:** Hemodialysis, Endotoxin, Water quality, Membrane permeability

## Abstract

**Background:**

Membranes with increasing pore size are introduced to enhance removal of large uremic toxins with regular hemodialysis. These membranes might theoretically have higher permeability for bacterial degradation products. In this paper, permeability for bacterial degradation products of membranes of comparable composition with different pore size was investigated with a new in vitro set-up that represents clinical flow and pressure conditions.

**Methods:**

Dialysis was simulated with an AK200 machine using a low-flux, high-flux, medium cut-off (MCO) or high cut-off (HCO) device (*n* = 6/type). A polyvinylpyrrolidone-solution (PVP) was recirculated at blood side. At dialysate side, a challenge solution containing a filtrated lysate of two water-borne bacteria (*Pseudomonas aeruginosa* and *Pelomononas saccharophila*) was infused in the dialysate flow (endotoxin ≥ 4EU/ml). Blood and dialysate flow were set at 400 and 500 ml/min for 60 min. PVP was sampled before (PVP_pre_) and after (PVP_post_) the experiment and dialysate after 5 and 55 min. Limulus Amebocyte Lysate (LAL) test was performed. Additionally, samples were incubated with a THP-1 cell line (24 h) and IL-1β levels were measured evaluating biological activity.

**Results:**

The LAL-assay confirmed presence of 9.5 ± 7.4 EU/ml at dialysate side. For none of the devices the LAL activity in PVP_pre_ vs. PVP_post_ was significantly different. Although more blood side PVP solutions had a detectable amount of endotoxin using a highly sensitive LAL assay in the more open vs traditional membranes, the permeability for endotoxins of the 4 tested dialysis membranes was not significantly different but the number of repeats is small. None of the PVP solutions induced IL-1β in the THP-1 assay.

**Conclusions:**

A realisitic in vitro dialysis was developed to assess membrane translocation of bacterial products. LAL activity on the blood side after endotoxin exposure did not change for all membranes. Also, none of the PVP_post_ solutions induced IL-1β in the THP-1 bio-assay.

## Background

Novel insights in uremic toxicity over the last decade have revealed that retention products in the middle molecular weight range 15–45 kDa might substantially contribute to the enhanced inflammation and cardiovascular mortality observed in patients with end stage kidney disease (ESKD) [1, 2]. Conventional high-flux dialyzers do not efficiently remove molecules in this molecular weight range, such as β2-microglobulin (β2M), tumor necrosis factor (TNF)-α, interleukins (ILs), and complement factor D, resulting in their accumulation in patients with ESKD [3]. The theoretical advantages of high-flux over low-flux membranes are not completely translated in clinical advantages, as demonstrated in the HEMO and MPO study [4–7]. Hemodiafiltration with postdilution does result in enhanced removal of these middle molecules [8], but also here, evidence to support their advantages in the clinical setting is conflictive [9–11], and part of the reported dose-response effect, with the largest effect observed in the subgroups with the highest substitution rates, might be due to confounding as patients in these subgroups typically have the best vascular access. In addition, hemo(dia)filtration increases technical complexity and cost of dialysis, and requires the application of high volumes of ultrapure dialysate and sterile substitution fluid with dedicated equipment.

Nowadays, there is a trend to further increase pore size and permeability of dialysis membranes to enhance removal of uremic toxins in the larger molecular weight range even when used in hemodialysis mode. The high cut-off (HCO) dialyzers allow elimination of molecules up to 45 kDa [12] and remove specific middle molecules more effectively than standard high flux membranes [13, 14]. The use of these membranes decreases inflammation and in vitro calcification, but also results in albumin loss [15, 16]. More recently, membranes with a steeper cut-off at a lower molecular weight level than the HCO membranes, the so-called medium cut-off (MCO) membranes, have been introduced [17]. These membranes can even remove large toxins such as kappa and lambda free light chains [18], two compounds associated with inflammatory markers and mortality in CKD [19–22]. In a recent randomized cross-over trial, use of MCO dialyzers during a 12 week study period was associated with a more expressed reduction of inflammation than the use of conventional high-flux dialyzers. However, in this study, ultrapure dialysate was used [23].

The question arises whether these membranes with increasing pore size also have higher permeability for endotoxins and other bacterial degradation products potentially present in dialysis fluids. This permeability issue is relevant since chronic exposure of hemodialysis (HD) patients to low levels of cytokine-inducing microbial components can potentially contribute to the micro-inflammatory status of these patients, thus neutralizing an eventual positive effect induced by their capacity of enhanced removal of pro-inflammatory uremic toxins [24, 25]. Therefore, the request for ultrapure dialysate might become a more important concern as membrane pore size becomes larger, even when applied in hemodialysis mode. The International Organization for Standardization (ISO) published the ISO11663:2014 which states that the dialysate should contain less than 100 colony forming units (CFU)/ml for bacteria and less than 0.5 endotoxin units (EU)/ml for endotoxin [26]. Standard methods to determine biological contamination of the dialysate are bacterial culture and the Limulus Amebocyte Lysate (LAL) assay. To test true biological response with more clinical relevance, bio-assays, such as the one using the THP-1 cell line, can be applied [27].

Assessment of endotoxin transfer over membranes has until now only been performed in closed in vitro dialysis circuits. [28–32]. In vitro investigations using miniaturized dialyzer modules of membranes with increasing pore size have indicated that endotoxin permeability with lipopolysaccharide (LPS) isolated from *E.coli* O55:B5 as a challenge and the LAL assay as read-out does not increase with increasing pore size [33]. However, none of these models used clinically relevant blood and/or dialysate flows, contamination exposure was rather high in most studies, and the biological response was assessed in biological assays using whole blood or isolated peripheral blood mononuclear cells which coincides with a need for healthy donors, large variability and lower sensitivity compared to the THP-1 assay.

In the present study a more advanced and realistic dialysis set-up using full sized dialysers was developed that simulates the clinical situation in terms of flow rates and viscosity of the medium perfused in the blood compartment, and using full sized dialyzers. This set-up was used to assess commercial dialyzers of comparable composition but with different pore size for their permeability for bacterial degradation products by means of a biological assay sensitive to several bacterial components as read-out in addition to the LAL assay.

## Methods

### Dialysis membranes

The dialysis membranes to be evaluated for their endotoxin permeability were provided by the manufacturer (Polyflux® 17 L, Revaclear 400, Theranova 400 and Theralite™ 2100, Baxter, Hechingen, Germany) and were composed of comparable polymers, but with a different pore size. Their main characteristics are summarized in Table 1.

**Table 1:** Characteristics of dialyzers

	Type	Sterilisation	Membrane Polymer	Effective Surface area (m^2^)	UF-coefficient (mL/H/mmHg)	Pore radius^a^ (nm)
Polyflux 17 L	Low flux	Steam	PAES/PVP/PA	1.7	12.5	3.1 ± 0.2
Revaclear 400	High flux	Steam	PAES/PVP	1.8	54.0	3.9 ± 0.1
Theranova 400	Medium cut off	Steam	PAES/PVP	1.7	48.0	5.0 ± 0.1
Theralite 2100	High cut off	Steam	PAES/PVP	2.1	52.0	10 ± 2

*PAES* polyarylethersulfone, *PVP* polyvinylpyrrolidone, *PA* polyamide, *UF* ultrafiltration ^a^effective Stokes-Einstein radius: calculated from molecular weight cut-off measured with polydisperse Dextrane

### Dialysate and blood substitution fluids

Ultrapure dialysis fluid was prepared on-line with an AK200 dialysis machine (Gambro, Lund, Sweden) using a smartbag® (Fresenius Medical Care, Willebroek, Belgium) acid concentrate and a BiCart™ cartridge (Gambro) resulting in a dialysate containing 3 mM K^+^, 140 mM Na^+^, 1.25 mM Ca^2+^, 0.50 mM Mg^2+^ and 34 mM bicarbonate.

A 1.25% polyvinylpyrrolidone (PVP) (Luvitec® K85 powder, BASF, New Jersey, USA) solution was prepared in sterile PBS 10×, pH 7.2 (Gibco, Life technologies, Paisley, UK) and diluted 1:10 in sterile water (Braun, Melsungen, Germany) to achieve a solution with a kinematic viscosity of 4 mm^2^/s, to mimic the viscosity of whole blood [34]. Viscosity was verified with an Ubbelohde Capillary Viscometer avs310 (SCHOTT Instruments, Weilheim, Germany). For each experiment, 3 L of this solution was prepared and recirculated through the blood compartment of the dialyzer.

Compatibility of PVP dissolved in PBS (PVP_PBS_) with both the LAL-assay and THP-1 assay was evaluated per se and in combination with LPS in comparison to PBS. No interference of the PVP dissolved in PBS could be observed in both assays.

### Challenge solution

The ISO11663:2014 standard for LPS allows less than 0.5 EU/mL in dialysis fluid [26]. In the in vitro experimental set-up, the duration of a dialysis session was set to 1 h. Corresponding to the total exposure during a regular dialysis session of 4 h a minimum endotoxin load of 2 EU/ml should be aimed for. However, to create a worst case scenario, this load was increased, aiming at a dialysis fluid containing at least 4 EU/ml. To obtain this, a concentrated solution (200 EU/ml) of two clinically relevant water-borne bacterial species, *Pseudomonas aeruginosa* and *Pelomonas saccharophila* (BCCM/LMG, Gent, Belgium), was prepared [35, 36]. Both strains were cultured separately to a final concentration of 5.0 × 10^10^ CFU/ml for *P. aeruginosa* and 3.3 × 10^10^ CFU/ml for *P. saccharophila,* and harvested. The bacteria were treated with heat (20 min, 95 °C) followed by ultrasound (1 min, 10 rpm) to induce bacterial disintegration. The obtained lysates were combined as equal endotoxin units (65,000 EU of *P. aeruginosa* plus 65,000 EU of *P. saccharophila*) and diluted up to 50 ml with dialysis fluid to a total concentration of 2600 EU/ml. This solution was filtered [28–30, 32], with a Millex 33 mm Sterile Filter Unit with 0.45 μm pore size Durapore® membrane (Merck KGaA, Darmstadt, Germany) (1 filter per 10 ml) and further diluted up to 200 EU/ml with dialysis fluid. The solution was transferred to a sterile bag (Beldico, Marche-en-Famenne, Belgium) to be infused continuously into the dialysate circuit, aiming at a final concentration in the dialysate of 4 EU/ml.

### Dialysis machine set-up

The AK200 dialysis machine was set in double needle treatment and the tubings for hemodialysis (Gambro) and the dialyzers were connected. The different membranes were tested in random order as determined by an Excel based random generator; for each membrane type 6 different dialyzers were tested. The consecutive experimental steps are summarized in Table 2.

**Table 2:** Overview of the experimental steps of the in vitro dialysis

Experimental Step	Solution Blood side	Circulation blood side	Time (min)	Q_B_ (ml/min)	Q_D_ (ml/min)	Q_F_ (ml/min)	Infusion Contaminant
Priming Blood circuit	NaCl	Discard	5	100 ml/min	0	0	none
Priming Dialysate circuit	None	–	2	0	500	0	none
Exchange fluid in Blood circuit	Plasma	Discard	~2^a^	200	0	0	0
Coating	Plasma	Recirculate	40	200	0	0	0
Rinsing	PBS 1×	Discard	18	200	0	30	0
Exchange fluid in Blood circuit	PVP	Discard	~2^a^	200	0	0	0
Dialysis	PVP	Recirculate	60	400	500	0	10 ml/min

^a^time depends on volume of circuit + membrane. For Polyflux: 1′56″; Revaclear: 1′53″; Theranova: 1′52″; Theralite: 2′07″

First the blood circuit of the dialyzer was primed with 0.9% NaCl (Clear-flex, Baxter, Lessen, Belgium), followed by further priming of the dialysate circuit with ultrapure dialysis fluid. After discarding the priming solution, 1 L of human plasma (Octaplas, Octapharma, Langenfeld, Germany) was recirculated through the blood circuit side of the dialyzer with a bloodflow rate (Q_B_) of 200 ml/min at 37 °C for 40 min with continuous mixing in the reservoir on a magnetic stirrer, in order to coat the membrane with plasma proteins [31, 37, 38]. During this procedure, the dialysate circuit was sealed off (dialysate flow rate (Q_D_) = 0 ml/min) and no ultrafiltration was allowed (ultrafiltration flow rate (Q_F_) = 0 ml/min). Afterwards the blood circuit was rinsed with PBS for 18 min at a Q_B_ of 200 ml/min in isolated filtration mode (ultrafiltration (UF) volume = 1.8 L/h). Then the PBS in the circuit was exchanged with the PVP solution.

**Fig. 1:**
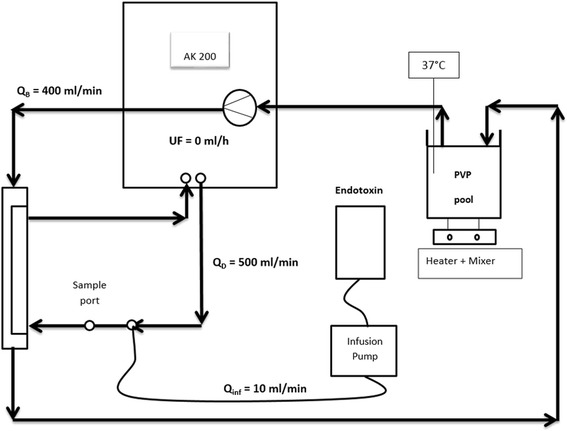
Schematic overview of the dialysis set-up. After priming both blood and dialysate circuit and after coating the test membrane with plasma, 3 L of PVP solution, continuously mixed, was recirculated at 37 °C at the blood side at a blood flow rate Q_B_ of 400 ml/min. Samples of the PVP were taken from the pool before and after the experiment. The dialysate was prepared by the AK200 and circulated at a flow Q_D_ of 500 ml/min. Before entering the membrane contaminant was infused at a flow Q_inf_ of 10 ml/min by means of a pump. Samples from the dialysate were taken just before the membrane at 5 and 55 min. Ultrafiltration Q_F_ was set at 0 ml/min

During the actual experiment 3 L of the PVP solution at 37 °C was recirculated during 60 min at a Q_B_ of 400 ml/min while the PVP pool was continuously mixed. The dialysate flow was set at 500 ml/min and the challenge solution was continuously infused from the sterile bag into the dialysate line before inlet of the dialyzer at a rate of 10 ml/min with a droplet pump (Cardinal Health, Brussels, Belgium), aiming at a contamination level of the dialysate above 4 EU/ml. A sampling port was placed between the contamination inlet port and the inlet of the dialyzer to assess the level of contamination. A schematic figure of the experimental set-up is shown in Fig. 1.

Samples of the dialysate were taken after 5 and 55 min. The PVP pool was sampled in duplicate before the start (PVP_start_) and at the end (PVP_post_) of the experiment. For the PVP_post_ solutions the LAL activity of the duplicates is reported separately, but the sample was considered positive for endotoxin if at least one contained a measurable endotoxin level. All samples were stored in pyrogen free glass tubes (Lonza, Walskersville, MD, USA). For the endotoxin quantification, samples were stored at 4 °C for a maximum of 24 h; for the cytokine induction assay samples were stored at −20 °C until analysis.

For decalcification and disinfection of the dialysis machine fluid path, a CleanCart C® (Gambro) cartridge was used in combination with a heat disinfection program after each experiment. For each experiment a new set of tubings was used.

### Endotoxin quantification

For quantification of intact LPS in the challenge solution, the dialysate and the PVP, the LAL test, a quantitative kinetic and chromogenic assay (Kinetic-QCL®) (Lonza), was used. The detection limit of this assay is 0.005 EU/ml. The test was applied according to the manufacturer’s guidelines.

### Cytokine induction assay with a THP-1 cell line

To evaluate the biological activity of potential contamination in the tested solutions, the THP-1 assay was used as described previously [27].The human monocytic cell line THP-1 (ATCC, LGC, Promochem, Middlesex, UK) was maintained as a continuous culture. Cell cultures at a density of 1 × 10^6^ cells/ml were differentiated with calcitriol (10 nM; Sigma-Aldrich, St Louis, MO, USA) for 72 h. Afterwards the medium was refreshed, followed by a 24 h rest period. The differentiated THP-1 cells were incubated in a 1:1 dilution (with a total volume of 700 μl) with the test solutions (dialysate, PVP_pre_ and PVP_post_) in polystyrene, pyrogen-free culture plates (Nunc, Roskilde, Denmark) for 24 h in a humidified atmosphere of 5% CO_2_ at 37 °C. A sample of the cell culture medium was included as a negative control and 25 EU/ml LPS (*E. coli* 055:B5; Lonza) in PBS was included as a positive control. After incubation, the culture suspensions were collected and stored at −20 °C. After a centrifugation step (5 min, 3000 rpm) IL-1β was quantified in the culture supernatant using a sandwich ELISA kit (Quantikine, R&D Systems, Abingdon, UK) according to manufacturer’s guidelines.

### Logarithmic retention value

As a measure of endotoxin retention capacity the logarithmic retention value (LRV) for each of the membranes was calculated using the following equation:

$$ LRV={\log}_{10}\frac{Endotoxin\ \frac{Dialysate_{5\prime }+{Dialysate}_{55\prime }}{2}}{Endotoxin\ {PVP}_{post}} $$

### Statistics

Data are expressed as mean ± SD. Measurements below the limit of detection of the LAL-assay were replaced by the LOD/√2 [39]. Statistical analysis was performed using a One-way ANOVA or an unpaired T-test using GraphPad Prism 4.0. A *p*-value of less than 0.05 was considered significant.

## Results

### Assay compatibility of PVP

PVP dissolved in PBS (PVP_PBS_) at a concentration of 12.5 g/L was evaluated for its possible interference with both the LAL-assay and the THP-1 assay. In the LAL-assay, PBS and PVP_PBS_ were tested per se and as a dose response with LPS in PBS and PVP_PBS_. The LPS retrieved with the LAL-assay was within the acceptable range of 50%–200% of the added concentration (data not shown) [40]. In the THP-1 assay, PBS and PVP_PBS_ were evaluated per se and in combination with 45 EU/ ml LPS. THP-1 cells did not produce IL-1β in the presence of PBS and PVP_PBS_, but when LPS was added the cytokine expression was comparable to the response of LPS dissolved in PBS as such (data not shown). Thus the combination of PVP dissolved in PBS does neither interfere with the results of the LAL nor with the THP-1 assay.

### Permeability of dialysis membranes for bacterial filtrates

Table 3 shows the individual and the mean LAL assay responses for the dialysate and PVP solutions per individual experiment, categorized per membrane.

**Table 3:** Endotoxin levels in the dialysate and the PVP solution per membrane measured by the LAL-assay (*n* = 6 for each membrane). LOD was 0.005 EU/ml

Membrane	Dialysate (EU/ml)	PVP_pre_ (EU/ml)	PVP_post_ (EU/ml)		Statistics PVP_pre_ vs PVP_post_
			Duplicate 1	Duplicate 2	
Low-flux	3.5	< LOD	< LOD	< LOD	
	3.9	< LOD	< LOD	< LOD	
	5.5	< LOD	< LOD	< LOD	
	9.4	< LOD	< LOD	< LOD	
	10.3	< LOD	***0.011***	< LOD	
	19.1	< LOD	< LOD	< LOD	n.s.
Mean ± SD	**8.6 ± 5.8**				
High-flux	3.6	< LOD	< LOD	< LOD	
	4.1	*0.008*	*0.007*	*0.008*	
	5.9	< LOD	***0.005***	< LOD	
	6.1	< LOD	< LOD	< LOD	
	20.0	*0.007*	< LOD	< LOD	
	33.7	< LOD	< LOD	< LOD	n.s.
Mean ± SD	**12.2 ± 12.2**				
Medium cut-off	6.0	< LOD	***0.023***	***0.005***	
	6.3	< LOD	< LOD	< LOD	
	6.8	< LOD	< LOD	< LOD	
	8.0	< LOD	***0.006***	***0.005***	
	10.6	< LOD	***0.005***	***0.005***	
	11.8	< LOD	< LOD	< LOD	n.s.
Mean ± SD	**8.3 ± 2.4**				
High cut-off	3.2	< LOD	***0.019***	***0.013***	
	4.1	< LOD	***0.005***	***0.005***	
	5.4	< LOD	***0.005***	***0.005***	
	5.8	< LOD	***0.005***	< LOD	
	12.1	< LOD	< LOD	< LOD	
	22.5	*0.006*	*0.005*	*0.005*	n.s.
Mean ± SD	**8.9 ± 7.4**				

Data with measurable endotoxin levels were written in italic and when they were higher than the PVPpre value they were additionally put in bold and underlined

Although dialysate-endotoxin concentration varied between 3.2 and 33.7 EU/ml in the individual experiments, mainly due to the difficulty of filtrate preparation and complexity of the experimental set-up, the mean exposure to endotoxins through the contaminated dialysate was above the intended minimum 4 EU/ml for each of the different experiments and did not differ between the different membranes. No correlation was found between endotoxin load and permeability.

LAL activity in the PVP solution at the blood side of the dialyzer was below the limit of detection (LOD = 0.005 EU/ml) both before (PVP_pre_) and after the experiment (PVP_post_) for 12 out of 24 tested dialyzers. Endotoxin levels were below LOD in all but three PVP_pre_ solutions. This potentially indicates contamination occurred already before the start of the experiment in these three experiments. Positive PVP_post_ reading higher than the corresponding PVP_pre_ reading, indicating possible contamination from endotoxin in the dialysate, was found in 9 out of 24 experiments (low-flux: 1/6; high-flux: 1/6; MCO 3/6; HCO: 4/6) however, there was no apparent correlation between the individual endotoxin challenge concentrations and the detectable readings and in three of these cases only one of the duplicate tests was positive.

### Logarithmic retention value

Using the LAL-data as measured above, the LRV values vary from 3.09 ± 0.50 for HCO membranes over 3.16 ± 0.29 and 3.21 ± 0.28 and for low-flux and MCO membranes respectively to 3.29 ± 0.46 for the high flux membranes. No statistical differences were found between the four membrane types.

### Cytokine induction assay

As shown in Table 4, 25 EU/ml LPS and the contaminated dialysate significantly induced IL-1β expression, whereas none of the PVP solutions used in the different experiments induced IL-1β expression neither before or at the end of the experiments. Moreover, no significant difference in induction of IL-1β expression was found between the PVP solutions treated with the different membranes.

**Table 4:** Overview of IL-1β expression in pg/ml in the THP-1 cytokine induction assay by the dialysate and PVP solutions

	Medium	LPS 25EU/ml	Dialysate	PVP_pre_	PVP_post_	Statistics^°^
Low-flux	11.44 ± 4.05	53.78 ± 21.11*	51.69 ± 57.07	10.87 ± 4.02	10.53 ± 4.22	n.s.
High-flux	12.86 ± 3.43	62.03 ± 22.91*	88.4 ± 122.13	11.87 ± 3.09	11.09 ± 2.82	n.s.
Medium cut-off	11.93 ± 3.54	54.71 ± 20.85*	40.99 ± 60.49	12.11 ± 3.55	11.13 ± 2.87	n.s.
High cut-off	12.25 ± 3.69	59.97 ± 17.22*	22.78 ± 21.88	11.55 ± 3.69	11.30 ± 3.18	n.s.

^*^ *p* < 0.05 vs Medium; °PVPpre vs. PVPpost

## Discussion

In the present study a new, clinically more representative model to test permeability of dialyzers for endotoxins is demonstrated. It is to our knowledge the first time that permeability of membranes was tested using a relevant, realistic set-up mimicking real life settings and conditions. Using this set-up, four dialysis membranes of comparable composition but with different pore size were tested for their permeability for endotoxins by exposing them during a 1 h in vitro dialysis session to dialysate contaminated with filtrates of two water-borne bacteria, *P. aeruginosa* and *P. saccharophila*, at an endotoxin challenge at least four times the upper limit of endotoxin load (2 EU/ml) when using standard dialysis fluid [26]. For the tested membranes, there was a non-significant difference in number of the PVP solutions which contained a detectable amount of endotoxin after repetitive circulation through the dialyzer, be it close to the detection limit in the majority of cases. It was chosen to give the individual data from all experiments as this is the most exact way to present the data and to give the reader full visibility of the data. The PVP solutions in many cases had a measured LAL activity below the LOD and the individual numbers give a better impression of the limited degree of contamination in case values were above LOD in the blood compartment. It might be speculated that increasing the number of experimental repeats could lead to statistical significance in the differences between the membranes, however this will not change the clinical relevance of the low degree of contamination. Interestingly, there was no dose-response correlation between the level of contamination within the tested range of dialysate endotoxin concentration and the detectable concentration of endotoxin in the PVP_post_ solutions, neither across the whole data set (all tested dialyzers) nor in any of the individual data sets (the four membranes tested). None of the PVP solutions induced measurable IL-1β expression in the THP-1 assay, and there was no difference in the logarithmic retention values as based on the measured LAL-levels, and for none of the devices the LAL activity changed significantly PVPpost vs. PVPpre.

Over the last years, membranes with larger pore sizes, such as MCO and HCO, have been introduced in an attempt to reduce inflammation and cardiovascular toxicity in patients with ESKD by an enhanced clearance of middle molecular weight substances during dialysis. Several trials have meanwhile provided evidence suggesting that larger pore membranes indeed enhance clearance of different middle molecules, and can reduce inflammation and calcification. A study in hemodialysis patients treated with HCO vs. high-flux membranes demonstrated by exposing THP-1 monocytes their serum or to the dialysate that HCO membranes eliminate a spectrum of pro-inflammatory mediators from serum into the dialysate [41]. In patients with signs of chronic mild inflammation (C-reactive protein (CRP) > 5 mg/l), use of HCO in series with a low-flux dialyzer resulted in a dampening of systemic inflammation markers such as soluble TNF receptor 1 (sTNFR1), associated with (cardiovascular) outcomes in observational CKD cohorts [42], but not of expression density of the P-selectin receptor CD162 on monocytes in pre-dialysis blood samples [15]. In an unblinded randomized cross over study comparing hemodialysis of equal duration using a high-flux vs. an MCO membrane during 4 weeks each, pre-dialysis TNF-α and IL-6 mRNA expression levels in white blood cells were reduced when using MCO compared to conventional high flux, but no effect was observed on CRP levels [23]. In addition, TNFR1 and kappa and lambda free light chains serum levels were reduced more effectively by MCO. Unfortunately, these studies did not assess actual removal of these substances, so it remains unclear whether the effects were due to reduced production or to enhanced clearance of the retention products. Also, it is remarkable that the effect of using larger pores only influenced levels of some and not all cytokines, despite them having the same range of molecular weight. This might point to differences in distribution volume, or in generation rate.

These studies, while promising, still refer to surrogate markers assessed. So far, no study of the impact of MCO or HCO membranes on patient relevant (hard) endpoints, such as mortality, cardiovascular events or even quality of life has been performed. The clinical benefits of the use of MCO and HCO, while promising, thus still need further underpinning.

Upregulation of production of cytokines by the use of the larger pore size membranes might be of concern when their usage would result in pro-inflammatory factors by transmigration of contaminants from the dialysate side. So far, all of the above mentioned studies have been performed in a setting where ultrapure dialysate was used. If the use of larger pore membranes would result in a substantial transmembrane transport of bacterial degradation products in settings with poor water quality, the positive effects of this type of membranes on inflammation could potentially be completely annihilated by the added inflammatory burden induced by these translocated contaminants. A case-study by Gong et al. reported a risk for endotoxemia when using an HCO membrane in combination with conventional dialysis fluid (0.112–0.141 EU/ml) [43]. However these results rely on a single measurement and as reviewed by Wong et al. caution should be taken when measuring endotoxin levels in blood using the LAL assay as its sensitivity and specificity for biological samples is poor [44]. The present experiments demonstrate that, when using a 4-fold overload of endotoxin, the use of larger pore membranes, MCO and HCO, is likely safe from that regard. Indeed, in none of the experiments, biological activation of the inflammatory system was observed as measured by a IL-1β production by the THP1-assay, sensitive for several bacterial components such as intact LPS, LPS fragments, peptidoglycan and short bacterial DNA fragments [27]. In the majority of experiments, no measurable transmembrane migration of endotoxin took place. The highest measured level of endotoxin in a duplicate sample from the ‘patient side’ was 0.023 EU/ml. This corresponds to a total amount of about 70 EU transferred during the 1 h dialysis session, and thus a transfer rate of about 1 EU/kg/h (mean patient of 70 kg), which is still well below the pyrogenicity limit of 5 EU/h/kg body weight (the minimum dose that induces fever) for injectable medications and devices [26].

Of note, our experimental set-up is a worst case scenario in which endotoxin exposure is 4-fold greater than permitted in standard (not ultrapure!) dialysate, spread over a 4 h dialysis session. It is of note that most modern dialysis monitors have an additional ultrafilter between permeate and dialysate, providing an extra safeguard for contamination that was bypassed in our set-up infusing the contaminant at the dialyzer inlet. Our results demonstrate that in facilities where water quality is within ISO standards of regular dialysate quality, the risk for cross-contamination of the blood side by endotoxins from the dialysate side when using membranes with larger pores is limited. In this type of setting, and by extension of course also in the setting of ultrapure dialysate, use of large pore membranes can potentially result in a dampening of chronic micro-inflammation. Larger long-term clinical trials with patient relevant outcomes are warranted to evaluate this hypothesis.

Special emphasis was made in the present study to develop an experimental model mimicking the clinical reality as close as possible. In the setting of the investigation of transmembrane migration of endotoxins from the dialysate to the blood side of the dialyzer, it is also important to keep in mind that this migration can not only take place by diffusion, but also by backfiltration. Backfiltration of contaminants can be influenced by properties of the membrane, such as pore size, surface area, and geometrical configuration of the fibers; by ultrafiltration rates, and thus by the hydraulic set-up of the dialysate circuit; by the viscosity of the solution circulating at the blood side of the membrane [45, 46]. Therefore, it was opted to use full size dialyzers rather than down-sized models, and these were assembled on a dialysis monitor as used in daily clinical practice, rather than using separate pumps to circulate the dialysate and the surrogate blood solution. Furthermore, to exclude confounding by differences in polymers, dialyzers of comparable membrane composition from a single manufacturer were chosen to focus on the investigation of variation in pore size. Therefore, the results of our investigation can likely not be generalized to membranes of different compositions or structure. The dialysate and blood flows were comparable to the ones applied in the clinic and in the same context, viscosity of the fluid circulating in the blood compartment was comparable to that of blood, mimicking clinical pressure distributions in the dialyzer and with it, realistic filtration profiles. To create a worst-case scenario ultrafiltration was set at 0 ml/min, resulting in a maximal backfiltration. It was opted not to use whole blood for the experiments since this is indeed cumbersome and expensive. In addition, concerns have been raised in the past that the use of whole blood in this type of experimental set up might abrogate activity of endotoxins as several components of whole blood have the capacity to neutralize endotoxins. The use of whole blood would thus potentially result in a false negative results when endotoxin transfer is being investigated [47].

Further, it was opted to apply a protein coating to the tested membranes by circulating a human plasma solution before the beginning of the experiments in order to mimic the properties of the synthetic membranes during dialysis, [37]. The differences in membrane properties after contact with blood can be attributed to the adhesion of proteins to the membrane surface, and is related to the membrane material [38, 48, 49]. A protein layer, also referred to as secondary layer, is created on top of the membrane due to the adhesion of proteins to the membrane surface, and acts also as a barrier for the transport of substances. It is known to be a process that is not instantaneous [49], but that occurs during the first hour of treatment [48]. This phenomenon cannot be described with the PVP model solution since the PVP molecules are unlikely to adsorb to the membrane surface. The coating is important with respect to biocompatibility in vivo [50], but it also decreases transport through the membrane and in this way also backfiltration of contaminants [37].

Finally, it is also important to use a relevant inoculum to create the endotoxin load. Sterile filtrates of *P. aeruginosa* and *P. saccharophila* were used as source of endotoxins, as they are relevant water-borne bacteria retrieved in dialysis circuits [36]. Preparations of bacteria can contain a wide range of contaminants and endotoxins with different molecular weight and thus different properties with regards to transmembrane transport. Not all of these bacterial products might test positive in the LAL test, but they do have a cytokine inducing capacity as assessed by the production of IL-1β by the THP-1 cell line in the bio-assay. Considering all these issues, the model presented here is very close to the clinical reality, adding more weight to the results and observations shown.

Remarkably, none of the PVP solutions induced a biological response as assessed by activity of human THP-1 monocytes higher than that of the background culture medium, despite the fact that in some of the PVP samples endotoxin was detectable. As demonstrated by Glorieux et al., the THP-1 assay only induces a significant increase in IL-1β expression in the presence of 0.1 ng/mL LPS (1.25EU/ml), comparable to the response of monocytes in healthy blood [27]. So based on the endotoxin levels quantified by the LAL-test, no induction of IL-1beta expression is to be expected. In contrast to the LAL-test, the THP1-assay reacts to more than endotoxin alone, suggesting that levels of other possible bacterial degradation products were also in the lower range.

## Conclusions

A realistic and feasible model to assess dialysis membrane translocation of bacterial degradation products present in the dialysate was developed and applied to test the retention capacity of 4 different membranes with similar chemical composition but different pore sizes. Although more blood side PVP solutions had a detectable amount of endotoxin using a highly sensitive LAL assay in the more open vs traditional membranes the permeability for endotoxins of the 4 tested dialysis membranes was not significantly different. Moreover, none of these PVP_post_ solutions induced IL-1β expression in the THP-1 based bio-assay that is sensitive also to other bacterial byproducts.

## Abbreviations

CFU: Colony forming units
CRP: C-reactive protein
ESKD: End stage kidney disease
EU: Endotoxin units
HCO: High cut-off
HD: Hemodialysis
IL-1β: Interleukin-1β
ISO: International Organization for Standardization
LAL: Limulus Amebocyte Lysate
LOD: Limit of detection
LPS: Lipopolysaccharide
LRV: Logarithmic retention volume
MCO: Medium cut-off
PVP: Polyvinylpyrrolidone
PVPPBS: PVP dissolved in PBS
PVPpost: PVP after the experiment
PVPpre: PVP before the experiment
QB: Blood flow rate
QD: Dialysate flow rate
QUF: Ultrafiltration flow rate
sTNFR1: Soluble TNF receptor 1
TNF-α: Tumor necrosis factor α
UF: Ultrafiltration
β2M: β2-microglobulin
